# Cost-effectiveness of a care manager collaborative care programme for patients with depression in primary care: 12-month economic evaluation of a pragmatic randomised controlled trial

**DOI:** 10.1186/s12962-021-00304-5

**Published:** 2021-08-17

**Authors:** Anna Holst, Frida Labori, Cecilia Björkelund, Dominique Hange, Irene Svenningsson, Eva-Lisa Petersson, Jeanette Westman, Christina Möller, Mikael Svensson

**Affiliations:** 1 Primary Health Care/School of Public Health and Community Medicine, Institute of Medicine, University of Gothenburg, Allmänmedicin, Box 453, 405 30 Gothenburg, Sweden; 2 Health Economics and Policy/School of Public Health and Community Medicine, Institute of Medicine, University of Gothenburg, Gothenburg, Sweden; 3 Region Västra Götaland, Närhälsan Research and Development Primary Health Care, Gothenburg, Sweden; 4 Division of Nursing, Department of Neurobiology, Care Sciences and Society, Karolinska Institute, Stockholm, Sweden; 5 Primary Health Care Head Office, Närhälsan, Region Västra Götaland, Gothenburg, Sweden

**Keywords:** Depression, Care manager, Cost-effectiveness, Primary care, Sweden, Collaborative care

## Abstract

**Objectives:**

To study the cost-effectiveness of a care manager organization for patients with mild to moderate depression in Swedish primary care in a 12-month perspective.

**Methods:**

Cost-effectiveness analysis of the care manager organization compared to care as usual (CAU) in a pragmatic cluster randomised controlled trial including 192 individuals in the care manager group and 184 in the CAU group. Cost-effectiveness was assessed from a health care and societal perspectives. Costs were assessed in relation to two different health outcome measures: depression free days (DFDs) and quality adjusted life years (QALYs).

**Results:**

At the 12-month follow-up, patients treated at the intervention Primary Care Centres (PCCs) with a care manager organization had larger health benefits than the group receiving usual care only at control PCCs. Mean QALY per patient was 0.73 (95% CI 0.7; 0.75) in the care manager group compared to 0.70 (95% CI 0.66; 0.73) in the CAU group. Mean DFDs was 203 (95% CI 178; 229) in the care manager group and 155 (95% CI 131; 179) in the CAU group. Further, from a societal perspective, care manager care was associated with a lower cost than care as usual, resulting in a dominant incremental cost-effectiveness ratio (ICER) for both QALYs and DFDs. From a health care perspective care manager care was related to a low cost per QALY (36,500 SEK / €3,379) and DFD (31 SEK/€3).

**Limitations:**

A limitation is the fact that QALY data was impaired by insufficient EQ-5D data for some patients.

**Conclusions:**

A care manager organization at the PCC to increase quality of care for patients with mild-moderate depression shows high health benefits, with no decay over time, and high cost-effectiveness both from a health care and a societal perspective.

*Trial registration details*: The trial was registered in ClinicalTrials.com (https://clinicaltrials.gov/ct2/show/NCT02378272) in 02/02/2015 with the registration number NCT02378272. The first patient was enrolled in 11/20/2014.

**Supplementary Information:**

The online version contains supplementary material available at 10.1186/s12962-021-00304-5.

## Introduction

Depression is one of the most common health disorders [38]. The economic burden of depression disorder is substantial both for the affected individual and for society at large [16]. Health care costs, sick leave and early retirement account for the largest expenditures [15]. Depression causes 6% of the burden of all diseases in Europe in terms of disability adjusted life years (DALYs) [25]. During the Covid-19 outbreak in 2020, depressive symptoms have vastly increased in the populations [23]. In health care, where the needs increase faster than the human and financial resources, new evidence-based organizational methods and new treatments are required to provide the best possible care to as many people as possible.

The majority of people with depressive symptoms in Western countries seek treatment in primary care [6, [33]. In Sweden, approximately 15–20% of primary care patients suffer from depression or a depression-like state [30]. Around 70% obtain treatment in primary care [32], and only 20% of patients with depression are referred to specialist psychiatry [27]. However, guidelines have been based primarily on research in specialist psychiatry [38]. The WHO World Health Report 2008, entitled “Primary health care. Now more than ever” clearly articulates the need to mobilise knowledge on a primary care level [36] and most recently in the Swedish official report Good and close care—The right support for mental health [14].

Swedish primary care is to a large extent based on team work where a team of general practitioners, registered nurses, psychotherapists, physiotherapists and occupational therapists meets around the patient in a person-centered approach. Good accessibility and good continuity of care are important core values and the evidence is good that these strategies improve quality of care [4]. Coordination of patients' various healthcare efforts is another central function of primary care.

According to international studies, isolated measures such as increased screening for depression, educational efforts concerning diagnostics or strengthened psychological expertise do not increase the quality of care compared with care as usual (CAU) [10, 31]. Best international evidence for high quality care and effectiveness of treating depression supports collaborative care with a care manager [1, 12, 21, 22]. In the PRIM-CARE study, performed in Swedish primary care, the care manager organization in primary care was found to have positive effects for patients with depression regarding symptoms, remission, return to work and quality of life compared to CAU [5].

The care manager is the person at the primary care centre who is responsible for enabling patients with common mental disorders to receive high quality care. The care manager facilitates the patient’s understanding of his or her medical condition and participation in the design of a care plan, tailored to the needs of that individual. The care manager has continuous supportive contact, preferably via telephone, with the patient and follows up and evaluates symptoms and treatment in cooperation with other professionals involved in the patient’s treatment.

Apart from good clinical effects, there is strong evidence that this type of intervention is also cost-effective [37], although there are also studies showing admittedly good effects but at increased costs [7, 11]. The cost-effectiveness of a care manager organization in Swedish primary care in the PRIM-CARE study proved to be good when examined in a 6-month perspective. The cost per QALY gained was from a health care perspective 67,731 SEK (€6 773) and from a societal perspective the care manager organization was dominant. [19]. However, it is important to study long-term effects of interventions. Therefore, this article presents the results from the 12-month follow-up of the PRIM-CARE study.

## Methods

### Intervention

#### PRIM-CARE

The present study is based on primary data gathered from the pragmatic cluster RCT PRIM-CARE using PCCs as the level of randomisation (project Clinical Trials NCT02378272) [5]. It can be viewed as a pragmatic (randomised controlled) effectiveness trial, which is generally regarded as the best vehicle for cost-effectiveness analysis (CEA) [24].

The PRIM-CARE study was performed at 23 Swedish PCCs in the regions Västra Götaland and Dalarna between December 2014 and January 2016 and included 376 patients, recently diagnosed with mild to moderate depression (< 1 month, according to PRIME-MD [26] and MADRS-S < 35 [20]). The GPs and the nurses at the PCCs were instructed to invite all patients, eighteen years or older, with mild to moderate depression, except from patients with severe psychiatric disorder, suicidal ideation or anamnesis of suicidal attempt, cognitive disability, substance abuse or insufficient knowledge of the Swedish language. The PCCs were randomised into two groups: intervention (n = 11) and control (n = 12), where intervention patients (n = 192) had care manager contact for 3 months and control patients (n = 184) received CAU. The primary outcomes were patients’ depressive symptoms measured by MADRS-S and Beck Depression Inventory II (BDI-II) [2] and patients' quality of life (assessed by EuroQoL-5D 3L scale [35] (weighted UK time trade-off values)). Secondary outcomes were sick leave days and return to work, service satisfaction, and antidepressant medication. Patients were assessed at baseline, 3, 6 and 12 months.

#### The care manager intervention

The intervention PCCs each established a nurse as care manager, who used 20–25% of her/his working time to coordinate and manage care and support of patients with depression [5]. Before the start of the trial, participating staff members were educated according to their tasks within the care manager programme i.e. 1 day for PCC directors, 2 days for general practitioners (GPs), 5 days for nurses/care managers. Programme services for participating patients included an individual care plan (1 h session per patient with care manager), standard phone contacts between care manager and patient for the assessment of self-rated depressive symptoms (at least 6–8 times during the 12-week intervention period), and the opportunity to contact the care manager at any unscheduled time if needed. In addition, care managers were in continuous contact with GPs, psychotherapists, and other health care personnel for updates about patients’ development. Thus, they did not perform any psychotherapeutic measures beyond behavioural activation but worked as a supportive connect between personnel and patients while improving accessibility and continuity of care, as well as treatment adherence.

Further, care managers had regular follow-up meetings (every second month) during the study, where both troubles and successful measures were discussed together with the research team and the region’s implementation team [29].

#### Care as usual

CAU could include visits to a GP, nurse, face-to-face psychotherapy (or being on the waiting list for such psychotherapy), antidepressants, sick listing, or combinations of these.

### Cost-effectiveness analysis (CEA)

#### CEA design

The cost-effectiveness of the care manager organization in the PRIM-CARE study was assessed compared to CAU. We take the standpoint from the Swedish health care and societal perspective. Further, we have a time-horizon of 12-months, and given the length of follow-up, we do not discount for future costs and health outcomes.

#### Health outcome metrics

The health outcome measures of the CEA were depression free days (DFD) based on depressive symptoms assessed by MADRS-S and quality adjusted life years (QALYs) based on EQ-5D-3L scores (weighted time trade-off values) assessed utilising the Dolan tariff [8]. The quantity of DFDs was assessed by estimating the number of days each patient scored equivalent to or below 12 on the MADRS-S. We use the cut-off value of ≥ 12 since score of 12 or below on MADRAS-S is considered as no depression or very mild depression. Taking into account that we have data at baseline, 3, 6 and 12 months, linear interpolation was carried out for study participants that had available data for all measure points, to predict a MADRS-S score for every day. The same linear interpolation used to calculate DFDs between the measurement points was also used for the EQ-5D-3L scores to be able to calculate the QALYs for every patient.

#### Cost measurements

Costs were estimated both from a health care and societal perspective. The health care perspective includes primary healthcare costs and medication costs. In addition, the societal perspective also considered cost due to productivity loss to primary health care and medication costs. We obtained the number of primary healthcare contacts from primary data collected via electronic patient records and patient research interviews from the RCT. Then we connected each primary health care contact to a unit cost according to market prices. Primary health care costs included education costs for PCC personnel (for the intervention group only) and visits within the primary health care to GPs, nurses, physiotherapist, and psychologists. All contact could be either face-to-face or via phone. Costs per primary health care contact and staff education were determined using the mean time spent and gross wages (including social fees) of the respective professional groups. All participating patients were followed-up concerning inpatient care; no one stated admittance to hospital care during the 12 months study period. Medicine used mainly consisted of antidepressants and was recorded per patient during the follow-up period, which was then connected to 2019 Swedish market prices [34]. Costs of productivity loss were calculated by means of the human capital approach [9], using enrolled sick leave days (percentagewise) during the follow-up period and the average gross wage (including social fees) for women in Sweden (since about two-thirds of the study population were female).

#### Statistical analysis

Due to missing responses of EQ-5D and MADRS-S at the different follow-ups, it was not possible to calculate QALYs and DFDs for all study participants. In the care manager group, EQ-5D data at all time points were available for 115 of 192 patients (60%), and DFDs data were available at all time points for 122 of 192 patients (64%). In the CAU group 128 of 184 (70%) had data for EQ-5D at all time points and 141 of 184 patients (77%) had data for DFDs at all time points (Additional file 1: Figure S1). To handle the missing values of EQ-5D and MADRS-S during the 12 months follow-up, we conducted a separate analysis where we carried out a multiple imputation of missing values of EQ-5D and MADRS-S. Missing values of EQ-5D and MADRS-S at 3-, 6- and 12-months follow-up were multiply imputed using chained nearest neighbour matching based on baseline EQ-5D, MADRS-S together with baseline characteristics concerning primary care centre, gender, number of children, marital status, country of birth, educational level, physical activity, tobacco use and medication.

Consequently, the results from the CEA are presented in a complete case analysis (CCA) approach and based on multiple imputation [3]. Patients were included in the CCA if costs, and EQ-5D and MADRS-S data were available at baseline, 3, 6 and 12 months follow-up (n = 230). We present the results from the CCA and multiple imputations in terms of an incremental cost-effectiveness ratio (ICER), which is the difference in costs divided by the difference in health outcome of implementing the care manager program compared to CAU: ICER = (Cost_care manager_ – Cost_CAU_)/(Health outcome_care manager_ – Health outcome_CAU_).

Sampling uncertainty was assessed using non-parametric bootstrapping carried out for the ICER on the cost per QALY with 1 000 bootstrap replicates. The result from the bootstrap was summarised in a cost-effectiveness acceptability curve (CEAC). The statistical analysis was carried out in Stata v.16.

#### Patient and public involvement

No patients took part in the development of the research question, outcome measures or in the recruitment of the study. Study participants will be informed about the results of the study via news media.

## Results

Table 1 presents the baseline characteristics for the intervention and control group. As expected, given the disorder, there were more women included the study. Mean age was around 41 years and a majority had employment but at study start, around 50% were on sick-leave. The average EQ-5D and MADRS-S score at baseline, 6 and 12 months for all available data is presented in Table 2.

**Table 1:** Descriptive statistics tabulated by treatment group

	All available data		Complete case sample	
	Care manager (n = 192)	Care as usual (n = 184)	Care manager (n = 105)	Care as usual (n = 125)
Age, mean (sd)	n = 192	n = 184		
Age in years at study start	41.2 (15.0)	41.9 (15.4)	44.2 (14.7)	43.2 (15.2)
Gender, n (%)	n = 192	n = 184		
Women	131 (68%)	137 (74%)	77 (73%)	99 (79%)
Men	61 (32%)	47 (26%)	28 (27%)	26 (21%)
Employment status, n (%)	n = 188	n = 184		
Working	137 (73%)	122 (66%)	77 (75%)	85 (68%)
Studying	18 (10%)	19 (10%)	6 (6%)	10 (8%)
Job-seeker	11 (6%)	13 (7%)	6 (6%)	8 (6%)
Other	22 (11%)	30 (16%)	13 (13%)	22 (18%)
Marital status, n (%)	n = 190	n = 184		
Not married	50 (26%)	43 (23%)	25 (24%)	31 (25%)
Married	61 (32%)	66 (36%)	37 (35%)	47 (37%)
Cohabitant	63 (33%)	57 (31%)	35 (33%)	37 (30%)
Divorced	13 (7%)	12 (7%)	7 (7%)	6 (5%)
Widow	3 (2%)	6 (3%)	1 (1%)	4 (3%)
Education level, n (%)	n = 191	n = 183		
Primary education	17 (9%)	27 (15%)	10 (10%)	14 (11%)
Secondary education	103 (54%)	90 (49%)	57 (54%)	60 (48%)
University	71 (37%)	66 (36%)	38 (36%)	51 (41%)
On sick-leave at baseline, n (%)	n = 184	n = 171		
Yes	93 (50%)	94 (55%)	50 (51%)	57 (49%)
Rate of sick-leave, n (%)	n = 92	n = 94		
100%	78 (85%)	84 (89%)	43 (86%)	51 (88%)
75%	2 (2%)	2 (2%)	0 (0%)	1 (2%)
50%	8 (9%)	4 (4%)	5 (10%)	2 (3%)
25%	4 (4%)	5 (5%)	2 (4%)	4 (7%)
Health status at baseline, mean (SD)	n = 192	n = 184		
MADRS-S	20.8 (7.2)	21.9 (7.1)	20.3 (7.0)	21.7 (6.5)
EQ5D-3L	0.58 (0.24)	0.56 (0.25)	0.57 (0.25)	0.56 (0.25)

**Table 2:** Mean EQ-5D and MADRS-S score of all available data

	Care manager (SD)	Care as usual (SD)
EQ-5D-3L		
Baseline	0.58 (0.24)	0.56 (0.25)
6 months	0.75 (0.18)	0.71 (0.22)
12 months	0.78 (0.19)	0.71 (0.27)
MADRS-S		
Baseline	20.8 (7.2)	21.9 (7.1)
6 months	10.3 (7.5)	13.3 (8.2)
12 months	10.6 (0.19)	13.2 (9.5)

### Cost outcome

Table 3 shows that the total healthcare cost for the care manager group was 6,037 SEK (€559) per patient versus 4,864 SEK (€450) for the CAU group during the 12-months follow-up (difference 1,173 SEK (€ 109), p-value 0.12). From the societal perspective, the mean cost of production loss (sick leave) added to healthcare cost resulted in a total cost of 111,974 SEK (€10,368) per patient in the care manager group during the 12-month follow-up. For the patients in the CAU group the corresponding costs were 150,861 SEK (€13,969) per patient during the 12-months follow-up. The difference in costs from the societal perspective was 38,887 SEK (€3,601) with p-value 0.26. However, neither of the differences in cost between the care manager group and CAU were statistically significant.

**Table 3:** Cost (Swedish kronor) and health outcome results, mean values (95% CI)

	Care manager (CM)	Care as usual (CAU)	Difference
Cost data (n = 376)			
Healthcare costs	6037 (5555–6519)	4864 (4393–5335)	1173 (− 329 to 2676)
Total costs	111,974 (87,795–136,153)	150,861 (119,291–182,430)	− 38,887 (− 108,850 to 31,077)
Health outcome data: complete case analysis (n = 230)			
QALYs	0.73 (0.70–0.75)	0.70 (0.66–0.73)	0.03* (− 0.005 to 0.07)
DFDs	203 (178–229)	155 (131–179)	37* (9–65)
Health outcome data: multiple imputation analysis (n = 376)			
QALYs	0.73 (0.71–0.75)	0.69 (0.66–0.71)	0.04* (0.002–0.08)
DFDs	206 (188–224)	165 (145–185)	33* (− 5 to 71)

*The difference is adjusted for the difference in baseline health-related quality of life and based on robust clustered standard errors. 1 Swedish krona (SEK) = €0.1

The largest share of health care costs were attributable to face-to-face and phone contacts with healthcare personnel (physician, nurse, psychotherapists, and physiotherapist). In the care manager group, these costs accounted for 75% of the healthcare costs and 85% in the CAU group. The care manager group had an additional cost for education of the staff in the PCC, which accounted for 15% of the health care costs. From a societal perspective, the cost of productivity loss was the largest contributor, accounting for 95% of the costs in the care manger group and 97% in the CAU group.

### Health outcome

Point estimates of the health benefits were higher in the care manager group compared to CAU group, regarding both QALY and DFDs, but only reached statistical significance with respect to DFDs in the complete case analysis (CCA) and with respect to QALYs in the multiple imputation analysis (although there were modest differences between the CCA and multiple imputation analysis). The mean EQ-5D-score increased in both groups at the 12-months follow-up. In the CCA, the mean QALY per patient was 0.73 in the care manager group compared to 0.70 in the CAU group (p = 0.086). The mean DFDs was 203 in the care manager group and 155 in the CAU group (p = 0.013). The results from the multiple imputation was similar to the CCA results, where the mean QALYs per patient was 0.73 versus 0.69 (p = 0.037) and the mean DFDs was 206 versus 165 (p = 0.088).

### Cost-effectiveness

As seen in Table 4 in the CCA, care manager was dominant compared to CAU at the 12- month follow-up, i.e. the care manager group gained more QALYs and DFDs to a lower cost than CAU, from a societal perspective. When applying the healthcare perspective, the cost per QALY was 36,500 SEK (€3,379) and the cost for one additional DFD was 31 SEK (€2.9). The results from the multiple imputation analysis were similar, with the care manager program being dominant from a societal perspective, and with a cost per QALY of 29,500 SEK (€2,731) and cost per DFD of 36 SEK (€3.3), respectively.

**Table 4:** Cost-effectiveness results

	Complete case analysis	Multiple imputation analysis
Healthcare perspective		
Cost per QALY	36,500 SEK/QALY	29,500 SEK/QALY
Cost per DFD	31 SEK/DFD	36 SEK/DFD
Societal perspective		
Cost per QALY	Care Manager Dominant	Care Manager Dominant
Cost per DFD	Care Manager Dominant	Care Manager Dominant

Care Manager Dominant = Care manager shows better health outcomes and lower costs. 1 SEK = €0.1

**Fig. 1:**
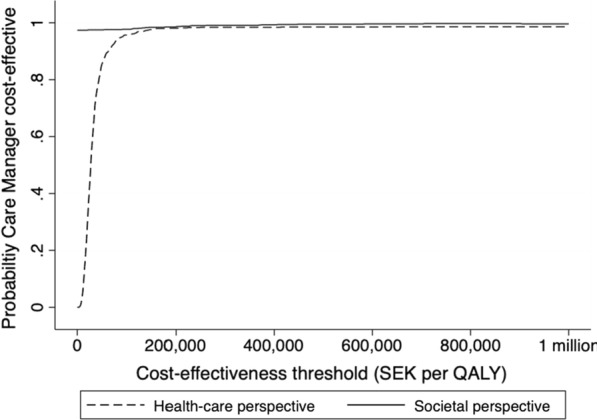
Cost-effectiveness acceptability curve (CEAC): health care and societal perspective in the 12-month evaluation of cost-effectiveness in the care manager collaborative care programme for patients with depression in primary care

Figure 1 presents the CEAC from a health care and societal perspective. The intervention had a high probability of being cost-effective at several threshold values per QALY. At a threshold value of 100,000 SEK per QALY, the probability of the intervention being cost-effective compared to CAU was 98%, and if the willingness to pay per QALY was 500,000 SEK, the probability of the intervention was cost-effective was 99.3%.

## Discussion

At the 12-month follow-up, patients with mild to moderate depression randomised to the care manager program at the primary care centre had larger health benefits than the group receiving usual care only. Further, from a societal perspective, the care manager organization was associated with a lower cost and better health outcomes than care as usual, resulting in the care manager organization dominating the CAU. This was shown also in the presentation of the 6-month follow-up results [19]. From a health care perspective the care manager organization was associated with a low cost per QALY (36,500 SEK / €3,379) and DFD (31 SEK/€3). This is to compare with the 6-month follow-up health care costs per QALY of 67,731 SEK (€6,773) and the cost per DFD of 67 SEK (€7). In the present analysis we can show, that the investment costs in education for PCC personnel for managing the collaborative care manager organization, being a single time investment for the PCC, were reduced over time and thus more cost-effective in a long-term evaluation.

The beneficial cost-effectiveness of the care manager organization at the PCC is probably a result of several effects. First, provider-patient communication with a person centred methodology, increased patient engagement in the depression care through behavioral activation and support. Second, by use of a self-assessment instrument with both pedagogic effects and follow-up possibility, and the establishment of long-term continuity of care, provided opportunities both for strengthening medication adherence and support for return-to-work. The principles of stepped care and team collaboration between GP, care manager, and psychotherapists at the PCC can be seen as a way of improving quality of care for the patient by facilitating communication between the patient and the care givers—and also between care givers with different competencies at the PCC resulting in cost-effective care at the patient, healthcare, and societal levels.

### Earlier literature

The cost-effectiveness of collaborative care organizations has been demonstrated earlier, although in most cases in shorter follow-up trials. Grochtdreis et al. concluded in their systematic review of 19 collaborative care cost-effectiveness analyses that using QALYs as effect measures with a time horizon of at least one year could further increase the evidence for cost-effectiveness of collaborative care for the treatment of depression disorders in primary care [17]. The high cost-effectiveness shown in the present study is primarily based on effects on the individual patient level both concerning the disorder—depression—and, most important—the secondary effect of more rapid and extensive recovery, i.e. earlier return to work for patients on sick leave. In the RCT of Goorden M et al. in the Netherlands, the positive effects of collaborative care, seen mostly from a societal perspective, were because of lower productivity losses due to absenteeism [13].

### Strengths and limitations

One of the strengths of the present study was the opportunity to compare the 6- and 12-month follow-ups, which yielded the most interesting finding i.e., the indication of a beneficial effect that did not decay over time. The difference in mean QALYs and DFDs per patient was larger at the 12-month follow-up compared to the 6-month follow-up [19]. Another strength is the scrupulous follow-up by electronic patient records during the 12 months concerning medication, sick leave certification and PCC contacts, both visits and telephone contacts. Further, the high participation rate also in the 12-month follow-up i.e. 70% (intervention group) and 79% (control group), supports validity of the results. Given the high concordance between both intervention and control group concerning baseline data and sick leave percentage status as well is an indication of representativeness of the included patient groups in a Swedish primary care context.

Among limitations is the fact that some patients that did not complete all four EQ-5D measurements. As in most “real world” pragmatic RCTs a 100% complete cases are a rarity. However, careful multiple imputation of missing values of EQ-5D scores were carried out to compensate for this, and showed results very similar to the complete case analysis, which could be an indication of the validity of the results.

The promising long-term results emphasise not only the positive effects of a care manager organization on patient level, but also, and perhaps more importantly, underline the advantage of complex interventions in the primary care context, where the complexity of depression illness today must be met with complex interventions adapted to the depressed person. This possibility should be present continuously during the entire course of the illness. Another benefit of the of the care manager model is the already shown high effectiveness of regular continuous contact based almost entirely on telephone contacts, an aspect of increasing importance during the current COVID-19 epidemic when healthcare should reduce face-to-face- physical contacts as much as possible.

It is also promising on a societal level that knowledge and personnel interventions in primary care for the wide group of individuals seeking care for mental health problems show high cost-effectiveness especially in the long run. In the PRIM-CARE study, interviews of the care managers indicated that the care manager task was gratifying and made their work meaningful, due to the possibility to achieve accessibility and continuity for the patient and to support and follow patients throughout the illness thus creating a safety net [28]. This indicates, that not only the patient, but also the personnel find the care manager organization at the PCC beneficial and that the care manager organization could indirectly also contribute to positive work environment and persistence among personnel [18]. This in turn would also reduce educational costs.

A final limitation that should be noted is that although we assess costs using a societal perspective where we include production loss, there are other potential societal considerations that we do not identify, such as potential effects on social services or the criminal justice sector, and we thus still have a limited societal perspective.

## Conclusions

A care manager organization at the PCC to increase quality of care for patients with mild-moderate depression shows high health benefits, with no decay over time, with even larger differences in mean QALYs and DFDs per patient at the 12-month follow-up compared to 6-month follow-up results compared to usual care. To invest in personnel education, being a once-for-all cost, has been shown to be an increasingly cost-effective measure in a long time perspective. The beneficial long-lasting effect for the patients is a strong incentive for wide implementation of a collaborative care organization with a care manager at the PCCs. The Swedish health care system should support this on a national level, to achieve a cost-effective and high quality care in primary care for common mental disorders.

## Supplementary Information

**Additional file 1**: **Figure S1**. Available EQ-5D and MADRS-S at each time point in the 12-month evaluation of cost-effectiveness in the care manager collaborative care programme for patients with depression in primary care.

## Data Availability

The datasets during and/or analysed during the current study available from the corresponding author on reasonable request.
