# Supplementary material for: Cost-effectiveness of a care manager collaborative care programme for patients with depression in primary care: 12-month economic evaluation of a pragmatic randomised controlled trial
Source: Cost Eff Resour Alloc. 2021 Aug 17;19:52. doi: 10.1186/s12962-021-00304-5 (PMC8369323; doi:10.1186/s12962-021-00304-5)
Supplement: Supplementary file 1 — Additional file 1: Figure S1. Available EQ-5D and MADRS-S at each time point in the 12-month evaluation of cost-effectiveness in the care manager collaborative care programme for patients with depression in primary care. [file 12962_2021_304_MOESM1_ESM.docx]

**Appendix Figure 1.** Available EQ-5D and MADRS-S at each time point in the 12-month evaluation of cost-effectiveness in the care manager collaborative care programme for patients with depression in primary care.
